# Posterior Tibial Neuropathy Secondary to Pseudoaneurysm of the Proximal Segment of the Anterior Tibial Artery with Delayed Onset

**DOI:** 10.1055/s-0038-1669403

**Published:** 2018-09-17

**Authors:** Abolfazl Rahimizadeh, Manuchehr Davaee, Majid Shariati, Shaghayegh Rahimizadeh

**Affiliations:** 1 Pars Advanced and Minimally Invasive Medical Manners Research Center, Iran University of Medical Sciences, Tehran, Iran

**Keywords:** anterior tibial artery, missile fragment, posterior tibial neuropathy

## Abstract

Anterior tibial artery is a nonvital artery which is one of the three arteries of the leg. This artery has a short proximal l segment in the popliteal region and a long segment in the anterior compartment of the leg designated as distal segment. With consideration of the deep location of the proximal segment in the popliteal fossa, it is less susceptible to trauma and subsequent formation of an aneurysm. On the contrary, the superficial long distal segment is more susceptible to trauma with high chance of pseudoaneurysm formation at the site of unrecognized injury. In this article, a 38-year-old military man being manifested about a decade after a trivial missile fragment injury with progressive posterior tibial neuropathy is presented. A giant pseudoaneurysm arising from the proximal segment of the anterior tibial artery was confirmed with angiography and the exact size of this pathology was documented with contrasted computed tomographic scan. The aneurysmal sac removal was accomplished after ligation of the corresponding artery proximal and distal to the sac followed by tibial nerve neurolysis which result in full recovery. In careful review we found that neither pseudoaneurysm arising from the proximal tibial artery nor posterior tibial neuropathy due to the compressive effect of the aneurysmal sac of this segment has been reported previously. Our primary purpose for reporting this case is not to describe the rarity of pseudoaneurysm formation at proximal segment of this artery but rather to describe delayed-onset posterior tibial vascular compressive neuropathy due to such an aneurysm. Eventually due to the potential sequel of a pseudoaneurysm, it is important for the surgeons to have high index of suspicion to prevent a missed or delayed diagnosis.

## Introduction

Arterial injuries are common event in military and civilian practice with iatrogenic one occurring in increasing frequency. 1 2 3 4

If the initial injury, in particular in noncritical arteries, is left unrecognized or is regarded insignificant to seek medical advice, a traumatic aneurysm might develop. 5 6 7 8 9 10 11

Traumatic aneurysms are true when the arterial wall injury occurs in intimal and medial layers of the artery with intact adventitia, and hence tend to be fusiform and their expansion is limited. A pseudoaneurysm or false aneurysm develops when all three layers of the artery are affected. In such circumstances, low-flow bleeding continues at the site of injury and this will gradually result in the reaction of the surrounding tissues with formation of a fibrous capsule around the hematoma. 7 8 9 10 11 This means that in a pseudoaneurysm, the hematoma freely communicates with the intravascular space, so that it can be designated as pulsating hematoma. For this reason, pseudoaneurysms are more likely to expand. With considerable expansion, adjacent structures and in particular the nearest traversing peripheral nerve might be compressed resulting in progressive neuropathy. 12 13

The leg arterial network is composed of three arteries: posterior tibial, peroneal, and anterior tibial. Anterior tibial artery is a nonvital artery of the leg with a short, but deeply located proximal and a long superficially located distal segment. Anterior tibial artery injury is one of the least among the arterial injuries of the extremities and account for approximately 2%, both in civilian and military experience. 1 5 6 8 Nonetheless, according to these cases series, vulnerability of the distal segment of this artery to trauma compared with its proximal segment becomes apparent. 14

On the contrary, formation of an arterial pseudoaneurysm due to missile fragment in the lower extremities and in particular in the leg is a well-known pathology in military practice. Reviewing the military experience since World War II, we found that traumatic aneurysms of anterior tibial artery account from 0 to approximately 2% of all traumatic aneurysms of the extremities with antipersonnel mines standing at the top. 1 5 6 8 15

Survey of civilian experience yielded the same result regarding the incidence. 4 11 15 16 17 18 19 20 21 In civilian communities, traumatic pseudoaneurysm of the anterior tibial artery is secondary to tibial or fibula shaft fractures, stab wounds, or low-velocity bullet injuries. 3 15 16 17 18 19 20 21

Recently, iatrogenic pseudoaneurysms of this artery due to orthopedic procedures are reported in increasing frequency. 22 23 24 25 26 27 28 29 30 31 32

In careful review of the literature, we found that the injury of the proximal segment of the anterior tibial artery has been reported only in one rare occasion, where not a single case of pseudoaneurysm originating from this segment has been reported. 14 This means that all published pseudoaneurysms of this artery have been confined to the distal segment.

In this article, we present the first example of a pseudoaneurysm of the proximal segment of the anterior tibial artery with compressive posterior tibial neuropathy which was diagnosed 12 years after being injured by a small missile fragment. This pathology was managed by exclusion of the pseudoaneurysm after ligation of the artery proximal and distal to the aneurysmal sac and subsequent neurolysis of the posterior tibial nerve.

Furthermore, where the usual delay in the diagnosis of the posttraumatic pseudoaneurysms of the extremities may range from a few days to several weeks with a mean of 45 days, onset of the pathology after such a long delay following a penetrating injury makes this case more interesting. 33 34 35 36 37

## Case Report

This 38-year-old military man was referred for the evaluation of radicular pain over the posterior aspect of his right leg and numbness at the planter aspect of his right foot for 3 weeks of duration. The patient had a history of being injured by several missile fragments 11 years before admission. With probable diagnosis of S1 root radiculopathy from L5–S1 disc herniation, lumbar myelography in another institution was normal. With continuing discomfort, he was referred to our institution.

**Fig. 1:**
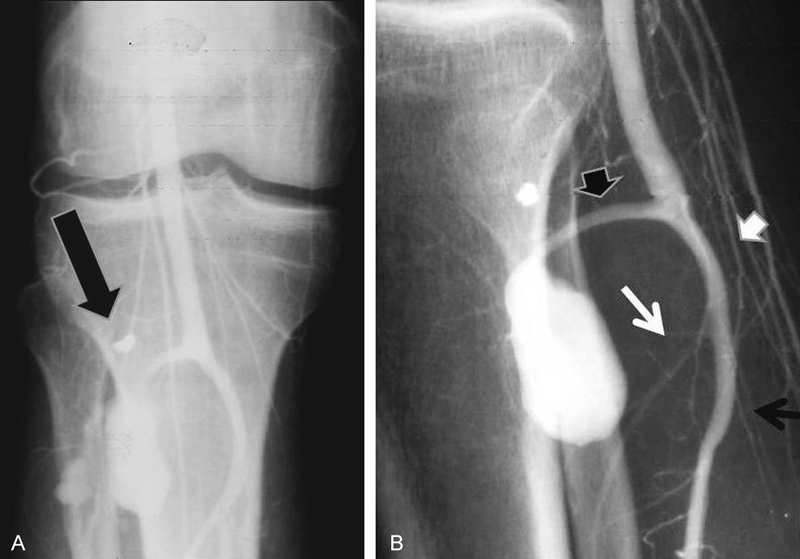
( **A** , **B** ) 0, AP and lateral femoral artery angiography show the pseudoaneurysm arising from proximal anterior tibial artery in popliteal region (black arrow head).The tibioperoneal trunk is patent (white arrow head). The posterior tibial artery (black arrow) is demonstrated where peroneal artery (white arrow seems to be narrow probably because of the compressive effect of the pseudoaneurysm. A small missile fragment is demonstrated in both views (short black arrows).

**Fig. 2:**
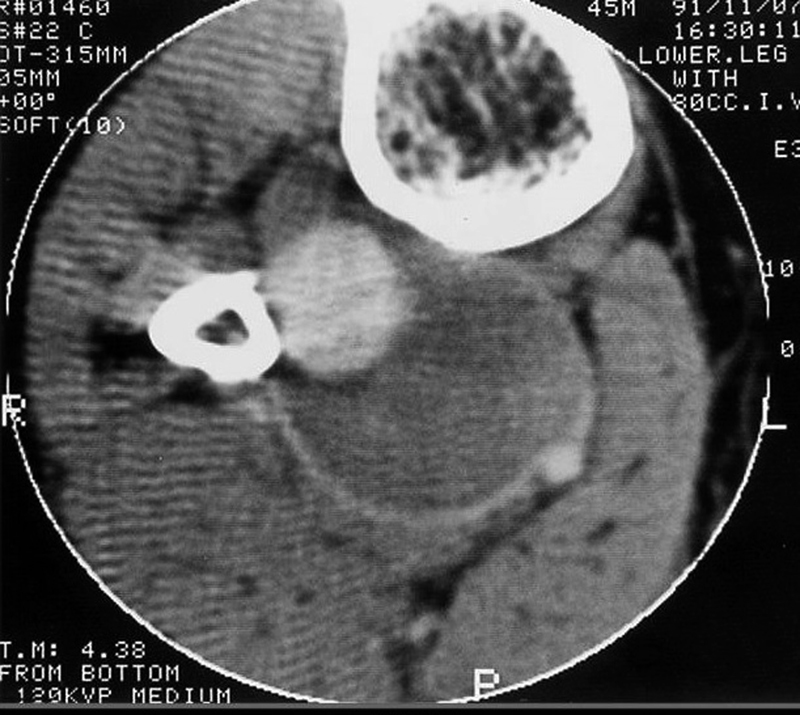
Contrasted CT scan showing the actual size of the aneurysm with its pseudocapsule being presented as rim enhancement. The patent part of the aneurysm is demonstrated as a hyperdense mass compatible with fresh blood which is surrounded by isodense area compatible with old clot. An artifact may be due to the missile fragment hidden behind the bone.

His neurological exam revealed distal sciatica at the course of S1 root, with hypoesthesia of the right sole. Further examination and palpation revealed a painful and pulsatile mass in the popliteal region. A bruit was heard in auscultation. With the diagnosis of a pseudoaneurysm, selective angiography was done and this revealed a pseudoaneurysm arising from the proximal segment of the anterior tibial artery. The artery bowed because of the compressive effect of the pseudoaneurysms ( Fig.1 ). With consideration of the existence of difference between the size of the aneurysm in angiography and the size of the mass in palpation, contrasted CT scan was done to estimate the exact size of the aneurysm. This showed a large isointense mass with rim enhancement surrounding a hyperdense area. The rim was compatible with the pseudocapsule of the aneurysm and the isointense mass was an old clot where hyperdense area was the patent part of the aneurysm ( Fig. 2 ).

## Intervention

The popliteal fossa was approached through a loose **S** -shape posterolateral incision. The popliteal, tibiofibular trunk and the proximal part of anterior tibial artery were identified and exposed. The proximal anterior tibial artery was ligated. Then the aneurysmal sac with dense fibrous wall was incised. Large quantities of old and fresh bloods were evacuated. Then, the orifice of the artery distal to the aneurysm was identified through its faint retrograde bleeding and this was sutured and occluded. Subsequently, the aneurysmal sac was dissected from surrounding structures including the flattened discolored posterior tibial nerve and was totally removed, and the affected nerve was released separately from the adhesions using magnification. Postoperative course was uneventful. At 2-month follow-up, he was neurologically normal.

## Discussion

Anterior tibial artery injury is one of the three arteries of the leg which has a deep proximal segment and superficial distal segment. Vulnerability of this artery to trauma differs in these two segments. For better understanding of this difference, review of its anatomy is necessary. This artery is the first branch of the popliteal artery which after traversing a few centimeters in the popliteal fossa pierces the interosseous membrane and descends into the anterior compartment of the leg along the shin in close proximity to peroneal nerve. The popliteal artery, currently being called tibiofibular trunk, divides into two branches, posterior tibial and peroneal arteries. Expectedly, the superficial and unprotected distal segment of the anterior tibial artery allows access for wounds not strong enough to be able to traumatize the short proximal segment embedded deeply in a rather more protected location. Even among the arteries of the popliteal region, the distal anterior tibial artery is the least susceptible. 14

With arterial injury left unrecognized, a pseudoaneurysm might develop. Especially if the initial trauma is regarded too insignificant to seek medical advice. 5 6 7 8 9 10 11 The pathogenesis of a pseudoaneurysm is characterized by a disruption of the arterial continuity with extravasation of blood into the surrounding tissues. 5 6 7 8 9 10 11 This leads to the formation of a fibrous sac that progressively enlarges due to the arterial pressure. 5 6 7 8 9 10 11

With more susceptibility of the distal segment of this artery, the pseudoaneurysms of this part of anterior tibial artery are reported in increasing frequency. In the past only missile wounds, low-velocity bullet injuries, penetrating injuries such as stab wounds, and tibia-fibula closed fractures had been the major causes of the pseudoaneurysms formation. 5 6 7 8 9 10 11 16 17 18 19 20 21 Nowadays, with the development of technology, iatrogenic etiology is the most frequent cause of this pathology, including percutaneous osteotomy, fixators, tibial nailing, locking plate, arthroscopy, meniscectomy, and knee and ankle arhtroplasty. 22 23 24 25 26 27 28 29 30 31 32

Before surveying the literature, with respect to the rarity of the distal segment arterial injury, a traumatic pseudoaneurysm developing at this site was expected to be an extremely rare event. Therefore, we were not surprised that not a single case of such pathology could be found in careful review.

The timeframe for the development or detection of a pseudoaneurysm after a trauma is quite variable and ranges from a few days to a few months or even years. In our case, the depth of the affected artery and the size of the missile fragment may explain the rather slow process and the unusual delay in diagnosis.

Pseudoaneurysm detection several years after injury has been reported only in six occasions including our case. 33 34 35 36 37 Surprisingly, three of these cases have followed trivial missile fragment injuries, one with gunshot wound 12 years earlier, one with history of stab wound, and the last one because of a nail puncture. 33 34 35 36 37

Usually, a peripheral nerve accompanies the arteries of the extremities and with formation of an aneurysm, vascular compressive neuropathy might appear. 12 13 The distal segment of the anterior tibial artery is in close proximity to the peroneal nerve and with sufficient enlargement of the corresponding aneurysm, peroneal nerve neuropathy might occur. 38

However, compressive neuropathy due to a pseudoaneurysm of proximal segment is unlikely because of its far distance from the nearest nerve, which is posterior tibial nerve. Reasonably, if posterior tibial compressive neuropathy coinciding a traumatic aneurysm of the proximal anterior tibial artery occurs, it should be an exception and only occur when the corresponding pseudoaneurysm reach to a giant size.

Historically, surgical strategy for pseudoaneurysm of less vital arteries had been ligation of the artery proximal and distal to the aneurysm followed by aneurysmal sac excision. 6 10 This remained the gold standard strategy for anterior tibial artery pseudoaneurysms. Actually, with classification of the anterior tibial artery as a nonvital artery, resection and re-establishment of continuity of this artery is regarded unnecessary. 20 However, preservation of the patency of the anterior tibial artery via autogenous vein graft is necessary only when the posterior tibial artery has been already occluded.

With improvements in medical technology, treatment options for pseudoaneurysms of the peripheral arteries have evolved to less invasive methods. 27 39 40 41 42 43 44 Advantages of these techniques are the ability to reach locations that would otherwise require extensive open surgical exploration. Furthermore, it allows rapid mobilization and return of the patient to normal activities. 27 39 40 41 42 43 44

These new therapeutic techniques were started with ultrasonic-guided manual compression of the neck of the aneurysm. Later, echo-guided thrombin injection was introduced which has been tried successfully in a patient with anterior tibial artery pseudoaneurysms. 40

Transcatheter coil embolization is another accepted treatment modality which offers many advantages including rapid safe occlusion and this had been successfully accomplished in the pseudoaneurysms of the anterior tibial artery as well. 40 41 In this procedure, occlusion should be performed both proximal and distal to the aneurysm for complete cessation of blood flow into and from the lesion.

Recently, endovascular anatomic reconstruction of the arterial wall with covered stent has been developed. 42 43 44 Eventually, this technique is not a vital procedure for anterior tibial artery pseudoaneurysms, although it has been utilized in a few rare instances.

Obviously, none of these noninvasive options are recommended in the patients with aneurysmal compressive neuropathy where excision of the aneurysm and subsequent neurolysis of the affected nerve are the mainstay of surgery. In our patient, because of posterior tibial neuropathy, open surgical intervention was preferred. Furthermore, reconstruction of the artery was not indicated because of the integrity of tibioperoneal trunk and its two major branches.

Duration and extent of compression have prognostic influence on associated neural recovery. 12 13 With early diagnosis, the neural function of the compressed nerve will recover soon after decompression.

In conclusion, we believe that this presentation can serve as a good reminder of another uncommon possible cause of posterior tibial neuropathy diagnosed with high index of suspicion. This report also emphasizes on the importance of taking into account the possibility of a pseudoaneurysm in differential diagnosis and a compressive neuropathy even years after trivial missile injuries.
